# Effect of Berberine on Glycation, Aldose Reductase Activity, and Oxidative Stress in the Lenses of Streptozotocin-Induced Diabetic Rats In Vivo—A Preliminary Study

**DOI:** 10.3390/ijms21124278

**Published:** 2020-06-16

**Authors:** Maria Zych, Weronika Wojnar, Magdalena Kielanowska, Joanna Folwarczna, Ilona Kaczmarczyk-Sedlak

**Affiliations:** 1 Department of Pharmacognosy and Phytochemistry, Faculty of Pharmaceutical Sciences in Sosnowiec, Medical University of Silesia, Katowice, Jagiellońska 4, 41-200 Sosnowiec, Poland; wwojnar@sum.edu.pl (W.W.); kielanowska.magdalena@gmail.com (M.K.); isedlak@sum.edu.pl (I.K.-S.); 2 Department of Pharmacology, Faculty of Pharmaceutical Sciences in Sosnowiec, Medical University of Silesia, Katowice, Jagiellońska 4, 41-200 Sosnowiec, Poland; jfolwarczna@sum.edu.pl

**Keywords:** berberine, diabetes mellitus, glycation, aldose reductase, oxidative stress, lenses, rats

## Abstract

Diabetes mellitus affects the eye lens, leading to cataract formation by glycation, osmotic stress, and oxidative stress. Berberine, an isoquinoline alkaloid, is a natural compound that has been reported to counteract all these pathological processes in various tissues and organs. The goal of this study was to evaluate whether berberine administered at a dose of 50 mg/kg by oral gavage for 28 days to rats with streptozotocin-induced diabetes reveals such effects on the biochemical parameters in the lenses. For this purpose, the following lenticular parameters were studied: concentrations of soluble protein, non-protein sulfhydryl groups (NPSH), advanced oxidation protein products (AOPP), advanced glycation end-products (AGEs), thiobarbituric acid reactive substances (TBARS), and activities of aldose reductase (AR), superoxide dismutase (SOD), catalase (CAT), and glutathione peroxidase (GPx). Diabetes induced unfavorable changes in the majority of the examined parameters. The administration of berberine resulted in an increased soluble protein level, decreased activity of AR, and lowered AOPP and AGEs levels. The results suggest that berberine administered orally positively affects the lenses of diabetic rats, and should be further examined with regard to its anticataract potential.

## 1. Introduction

Berberine is an isoquinoline alkaloid occurring predominantly in roots, rhizomes, and the stem bark of plants like *Hydrastis canadensis* L. and *Coptis chinensis* Franch. (family Ranunculaceae), *Phellodendron chinense* C.K.Schneid. (family Rutaceae), *Mahonia bealei* (Fort.) Carr., *Berberis aquifolium* Pursh, *Berberis vulgaris* L., and *Berberis aristata* DC. (family Berberidaceae) [1,2,3,4,5]. Berberin-containing plants have been being used since antiquity in traditional Chinese and Ayurvedic medicines, due to their antibacterial, antiprotozoal, and antidiarrheal properties [1,5]. The reports from recent years indicate that berberine reveals a broad range of pharmacological activities, including antidiabetic, hypolipemic, hypotensive, and anticancer ones. Berberine may also act as an antidepressant and anxiolytic, and can be used to prevent neurogenerative diseases [6,7,8,9,10,11].

Numerous scientific studies, based on in vitro and in vivo experiments conducted in various models, indicate that berberine regulates glucose metabolism via various mechanisms and signaling pathways [12,13]. Berberine promotes glycolysis by inhibiting mitochondrial respiratory chain complex I [14], inhibits gluconeogenesis in the liver [15], induces glycolysis in the peripheral tissues [16], promotes glucagon-like peptide 1 (GLP-1) secretion in the gastrointestinal tract [17], and activates glucose transport by glucose transporter 1 (GLUT1) [18]. It has been shown that this alkaloid can also modulate gut microbiome, which may prevent obesity and insulin resistance [19]. Berberine has been reported to be useful in type 2 diabetes treatment [12,13]. It reduces the levels of blood glucose and glycated hemoglobin, improves the lipid profile in the serum, reduces body mass, and enhances insulin sensitivity [20,21].

Even though there are reports indicating that berberine may protect from diabetes-related complications in the kidneys [22], heart [23], nervous system [24], or retina [25], there is no data as to whether berberine reveals any in vivo effect on diabetic complications in the lenses. It has been proven that antioxidants can delay development of ocular diseases, such as age-related macular degeneration, glaucoma, or cataracts [26,27]. Berberine is a hydroxyl radical scavenger, and its main metabolite—berberrubine—reveals even greater antioxidative activity than berberine [28]. It has been reported that after treatment with berberine, the levels of the oxidative stress-related markers decreased while the levels of endogenous antioxidants increased in the tissues of laboratory animals [29,30,31].

Attempts have been made to use berberine as an antimicrobial agent, in eye drops [32,33]. However, it should be noted that berberine has exerted a phototoxic effect in in vitro studies on the human retinal pigment epithelial cells. Thus, any eye-dedicated liquids containing berberine solutions or extracts prepared from plants containing berberine should be used cautiously when eyes can be exposed to bright sunlight. On the other hand, berberine-containing formulations probably do not cause phototoxicity in the eyes when administered orally [34]. It has been documented that berberine administered by gavage to mice protects the retinal photoreceptors from light-induced degeneration [35]. In in vitro studies, berberine has protected retinal pigment epithelium cells against H_2_O_2_-induced oxidative stress [36], attenuated apoptosis in rat retinal Müller cells stimulated with high glucose [37], and protected human retinal Müller cells from cytotoxicity induced by oxidized low-density lipoproteins (LDL) [38]. There is one report on an anticataract effect of berberine in vitro; the study was conducted on isolated goat lenses incubated in glucose [39]. To the best of our knowledge, there are no studies carried out in in vivo conditions in which the effect of berberine administered orally was evaluated with regard to the changes occurring in the lenses during diabetes. One of the well-established diabetes models that is used in laboratory rodents is a model involving streptozotocin as a diabetogenic agent [40]. This model allows, besides analyzing the changes in glycemia, examining diabetes-related alterations and diseases, such as cataracts at different time points, including the early phases [41,42,43].

The aim of this study was a preliminary evaluation of the effects of berberine on the changes in glycation products, aldose reductase activity, and oxidative stress-related markers in the lenses of rats with streptozotocin-induced diabetes in the period preceding the development of diabetic cataracts.

## 2. Results

**Figure 1:**
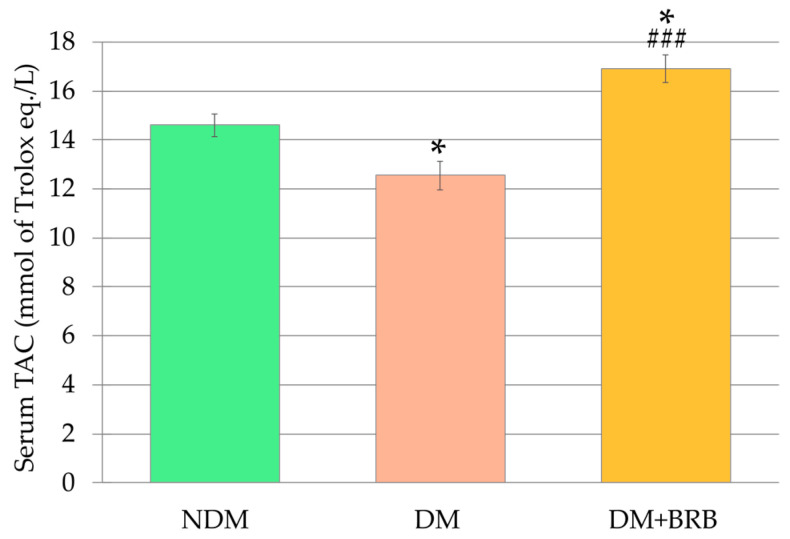
Effect of berberine administration to the diabetic rats on the total antioxidant capacity (TAC) in the serum. The results are presented as an arithmetical mean ± SEM. The statistical significance of the results was evaluated by ANOVA followed by Tukey’s honestly significant difference (HSD) post-hoc test. The results were considered statistically significant if *p* < 0.05: * *p* < 0.05—statistically significant difference in comparison with the NDM group; ^###^ *p* < 0.001—statistically significant difference between the DM + BRB and DM groups. NDM: non-diabetic control rats, DM: diabetic control rats, DM + BRB: diabetic rats treated with berberine at a dose of 50 mg/kg per os (p.o.) for four weeks.

In the control diabetic rats (group DM), the levels of non-fasting and fasting blood glucose were significantly higher 6 weeks after streptozotocin injection than in the control non-diabetic (not injected with streptozotocin) rats (group NDM). The body mass of the DM rats was significantly lower than the body mass of the NDM rats. Administration of berberine (BRB) at a dose of 50 mg/kg orally (per os—p.o.) for 4 weeks (group DM + BRB) did not affect any of these parameters, when compared to the DM rats. The total antioxidant capacity (TAC) in the serum of the DM rats was significantly lower than in the serum of the control NDM rats. Oral administration of berberine at a dose of 50 mg/kg for four weeks resulted in a statistically significant increase of TAC in the serum of the DM + BRB rats when compared to the TAC in the serum of the DM rats and the NDM rats (Figure 1).

### 2.1. Effect of Berberine Administration on Lens Mass and Soluble Protein Level in the Lenses

**Figure 2:**
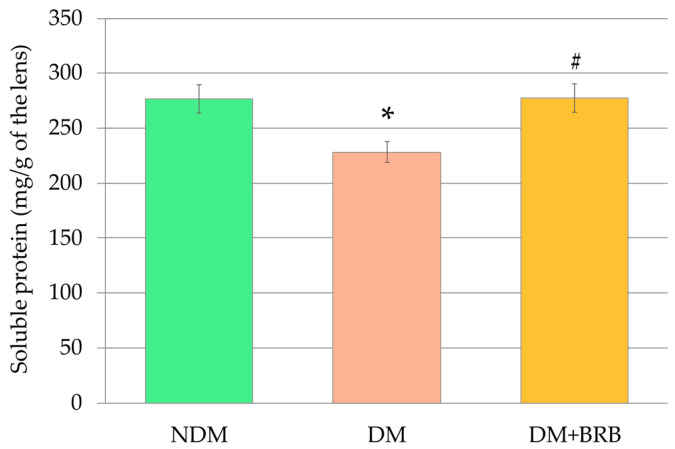
Effect of berberine administration to the diabetic rats on the soluble protein level in the lenses. The results are presented as an arithmetical mean ± SEM. The statistical significance of the results was evaluated by ANOVA followed by Tukey’s HSD post-hoc test. The results were considered statistically significant if *p* < 0.05: * *p* < 0.05—statistically significant difference in comparison with the NDM group; ^#^ *p* < 0.05—statistically significant difference between the DM + BRB and DM groups. NDM: non-diabetic control rats, DM: diabetic control rats, DM + BRB: diabetic rats treated with berberine at a dose of 50 mg/kg p.o. for four weeks.

The mean mass of the lenses of the control, non-diabetic (NDM) rats was 44.4 ± 1.0 mg. The mean mass of the lenses of the control diabetic rats (DM), as well as the lenses of the diabetic rats treated with berberine (DM + BRB), were not different from the mean mass of the lenses in the NDM group of rats (42.1 ± 1.0 mg for the DM rats, and 41.2 ± 0.9 mg for the DM + BRB rats). There were no differences in the mean lens mass between the DM and DM + BRB rats. The soluble protein level in the lenses of the DM rats was significantly lower than in the lenses of the NDM rats. Administration of berberine resulted in a significant increase of soluble protein level in the lenses of the DM + BRB rats compared with the lenses of the untreated DM rats. After berberine administration, the soluble protein level in the lenses of the DM + BRB rats did not differ from the soluble protein level in the lenses of the NDM rats (Figure 2).

### 2.2. Effect of Berberine Administration on the Advanced Glycation End-Products Level in the Lenses

**Figure 3:**
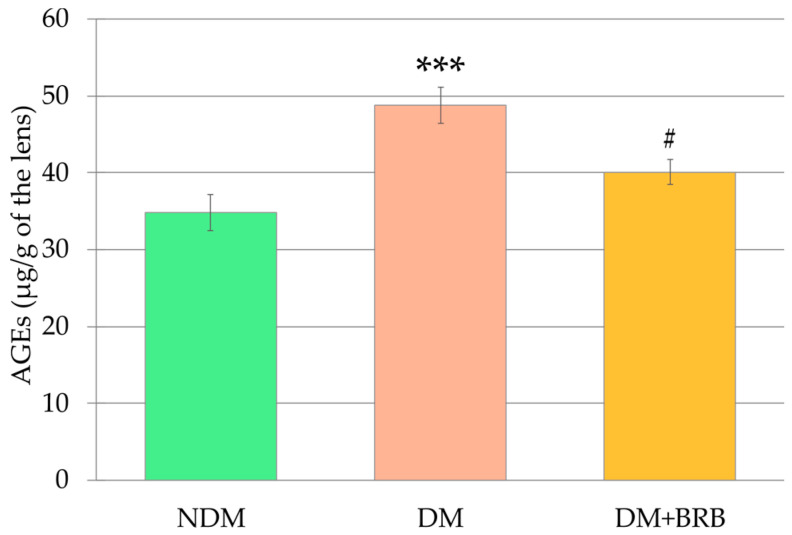
Effect of berberine administration to the diabetic rats on the level of advanced glycation end-products (AGEs) in the lenses. The results are presented as an arithmetical mean ± SEM. Statistical significance of the results was evaluated by ANOVA followed by Tukey’s HSD post-hoc test. The results were considered statistically significant if *p* < 0.05: *** *p* < 0.001—statistically significant difference in comparison with the NDM group; ^#^ *p* < 0.05—statistically significant difference between the DM + BRB and DM groups. NDM: non-diabetic control rats, DM: diabetic control rats, DM + BRB: diabetic rats treated with berberine at a dose of 50 mg/kg p.o. for four weeks.

The level of the advanced glycation end-products (AGEs) in the lenses of the DM rats was significantly higher than in the lenses of the NDM rats. Administration of berberine at a dose of 50 mg/kg p.o. to the diabetic rats for four weeks resulted in a significant decrease of the AGEs level in the lenses. As a result, the AGEs level in the lenses of the DM + BRB rats was not statistically different from the AGEs level in the lenses of the NDM rats (Figure 3).

### 2.3. Effect of Berberine Administration on the Aldose Reductase Activity in the Lenses

**Figure 4:**
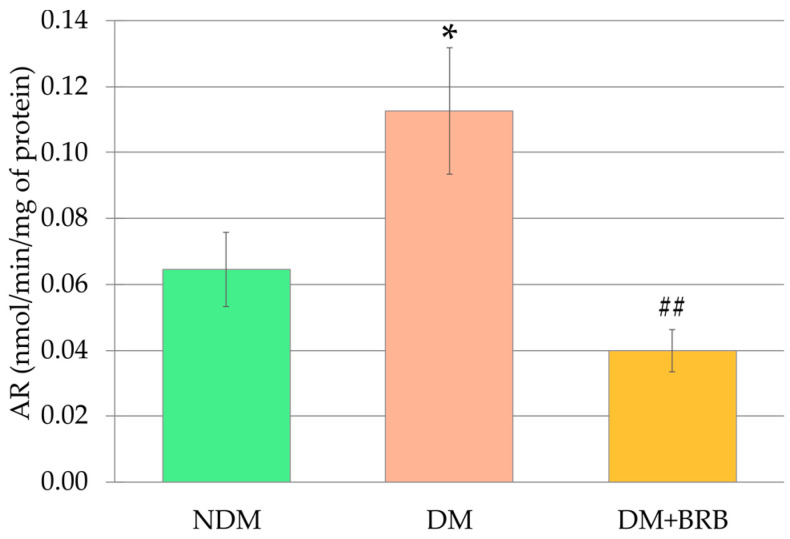
Effect of berberine administration to diabetic rats on aldose reductase (AR) activity in the lenses. The results are presented as an arithmetical mean ± SEM. The statistical significance of the results was evaluated by ANOVA followed by Tukey’s HSD post-hoc test. The results were considered statistically significant if *p* < 0.05: * *p* < 0.05—statistically significant difference in comparison with the NDM group; ^##^ *p* < 0.01—statistically significant difference between the DM + BRB and DM groups. NDM: non-diabetic control rats; DM: diabetic control rats; DM + BRB: diabetic rats treated with berberine at a dose of 50 mg/kg p.o. for four weeks.

The activity of the aldose reductase (AR) in the lenses of the untreated diabetic (DM) rats was significantly higher than in the lenses of the control rats (NDM). Administration of berberine at a dose of 50 mg/kg p.o. to the diabetic rats resulted in a significantly lower activity of this enzyme in the lenses of the DM + BRB rats than in the lenses of the DM rats. The AR activity in the lenses of the DM + BRB group of rats did not differ from the aldose reductase activity recorded in the lenses of the NDM rats (Figure 4).

### 2.4. Effect of Berberine Administration on the Activites of the Antioxidative Enzymes in the Lenses

**Figure 5:**
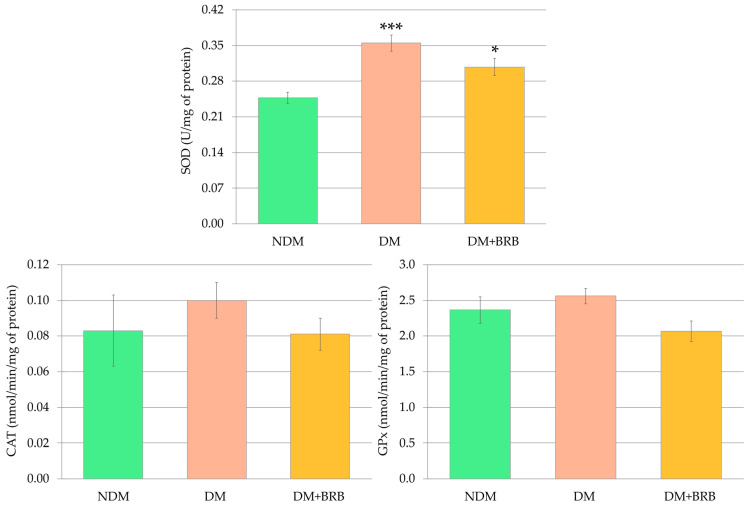
Effect of berberine administration to diabetic rats on the antioxidative enzyme activity in the lenses. The results are presented as an arithmetical mean ± SEM. The statistical significance of the results was evaluated by ANOVA followed by Tukey’s HSD post-hoc test. The results were considered statistically significant if *p* < 0.05: * *p* < 0.05; *** *p* < 0.001—statistically significant differences compared to the NDM group. NDM: non-diabetic control rats; DM: diabetic control rats; DM + BRB: diabetic rats treated with berberine at a dose of 50 mg/kg p.o. for four weeks; SOD: superoxide dismutase; CAT: catalase; GPx: glutathione peroxidase.

Diabetes resulted in an increased activity of superoxide dismutase (SOD) in the lenses of the DM rats compared to the SOD activity recorded in the lenses of the non-diabetic control (NDM) rats. Berberine administered to the diabetic rats at a dose of 50 mg/kg p.o. for four weeks did not affect the activity of this enzyme in the lenses of the DM + BRB rats compared to the DM rats. Moreover, the SOD activity in the lenses of the berberine-treated diabetic rats was significantly higher than in the lenses of the NDM rats. The activities of the other antioxidative enzymes—catalase (CAT) and glutathione peroxidase (GPx)—in the lenses of the DM group of rats were not changed in comparison with the lenses of the NDM rats. No statistically significant changes in the CAT and GPx activities were noted after berberine administration; therefore, the activity of these enzymes in the lenses of the DM + BRB rats was not statistically different from their activity in the lenses of either the NDM or DM rats (Figure 5).

### 2.5. Effect of Berberine Administration on the Non-Enzymatic Antioxidant Levels in the Lenses

**Figure 6:**
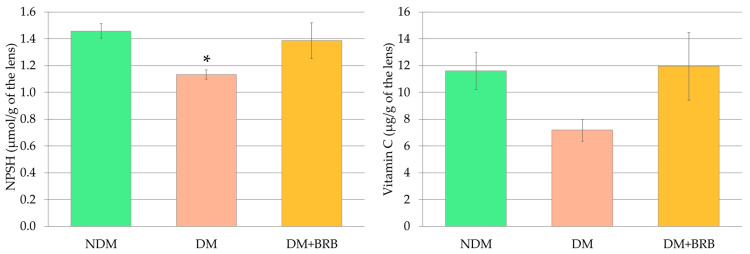
Effect of berberine administration to the diabetic rats on non-enzymatic antioxidant (NPSH and vitamin C) levels in the lenses. The results are presented as an arithmetical mean ± SEM. The statistical significance of the results was evaluated by ANOVA followed by Tukey’s HSD post-hoc test. The results were considered statistically significant if *p* < 0.05: * *p* < 0.05—statistically significant difference in comparison with the NDM group. NDM: non-diabetic control rats; DM: diabetic control rats; DM + BRB: diabetic rats treated with berberine at a dose of 50 mg/kg p.o. for four weeks; NPSH: non-protein sulfhydryl group.

In the lenses of the DM rats, a significant decrease in non-protein sulfhydryl group (NPSH) levels was observed. The NPSH levels in the lenses of the rats treated with berberine (DM + BRB rats) did not differ from the NPSH levels observed in the lenses of both the NDM and DM groups of rats. Similar yet statistically insignificant patterns of change were observed in vitamin C levels in the lenses of the rats subjected to this study (Figure 6).

### 2.6. Effect of Berberine Administration on Oxidative Damage Marker Levels in the Lenses

**Figure 7:**
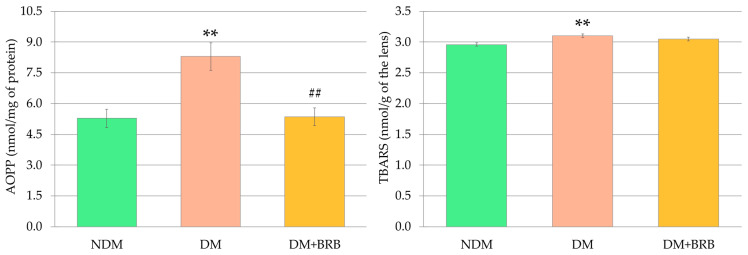
Effect of berberine administration to diabetic rats on non-enzymatic antioxidant (AOPP and TBARS) levels in the lenses. The results are presented as an arithmetical mean ± SEM. The statistical significance of the results was evaluated by ANOVA followed by Tukey’s HSD post-hoc test. The results were considered statistically significant if *p* < 0.05: ** *p* < 0.01—statistically significant differences in comparison with the NDM group; ^##^ *p* < 0.01—statistically significant difference between the DM + BRB and DM groups. NDM: non-diabetic control rats; DM: diabetic control rats; DM + BRB: diabetic rats treated with berberine at a dose of 50 mg/kg p.o. for four weeks; AOPP: advanced oxidation protein products; TBARS: thiobarbituric acid reactive substances.

In the lenses of the untreated, control diabetic (DM) rats, the levels of the markers depicting the damage to proteins and lipids were elevated in a statistically significant manner. The level of the advanced oxidation protein products (AOPP), a marker of protein damage, was significantly reduced in the lenses of diabetic rats after berberine administration. As a result of berberine action in the diabetic rats, the AOPP levels in the lenses of the DM + BRB rats were restored to the values observed for AOPP in the lenses of the NDM rats. On the other hand, the thiobarbituric acid reactive substances (TBARS) level, a marker describing the oxidative damage to lipids, was not affected by berberine administration, compared to the lenses of the untreated DM rats (Figure 7).

### 2.7. Principal Component Analysis

**Figure 8:**
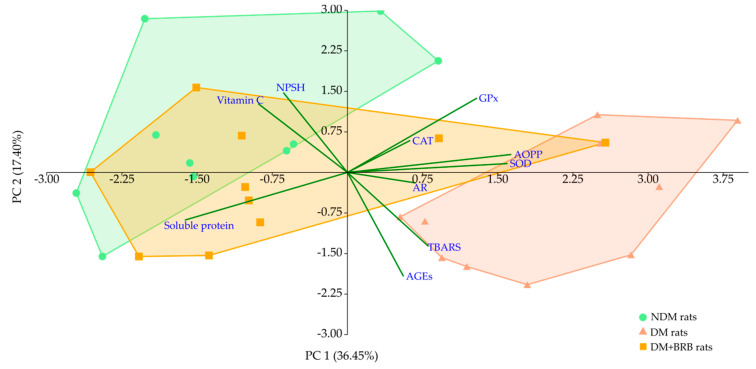
Principal component analysis (PCA) plot of biochemical parameters measured in the lenses. AGEs: advanced glycation end-products; AR: aldose reductase; SOD: superoxide dismutase; CAT: catalase; AOPP: advanced oxidation protein products; TBARS: thiobarbituric acid reactive substances; NPSH: non-protein sulfhydryl groups; PC 1: principal component 1; PC 2: principal component 2.

The principal component analysis (PCA) revealed a distinct clustering of experimental groups. A cluster formed by the NDM group separated significantly form the DM group cluster along both the principal components (PCs): PC 1 and PC 2. The cluster formed by the DM + BRB group shifted to the left part of the plot along PC 1 (which explained over 36% of total variability), and according to multivariate analysis of variance (MANOVA), it was separated significantly from the DM cluster and overlapped with the cluster formed by the NDM group. The main variables responsible for the NDM, DM, and DM + BRB cluster separation along PC 1 were the levels of soluble protein and AOPP, as well as the activities of SOD and GPx. The level of soluble protein positively correlated with the left part of the plot, and thus with the clusters formed by the NDM and DM + BRB groups, while the other main variables correlated positively with the cluster placed in the right side of the plot—i.e., the cluster of the DM group. Separation along the PC 2 axis (explaining 17.40% of total variability) resulted mainly from the AGEs, NPSH, TBARS, and vitamin C variables—higher values for NPSH and vitamin C correlated with the upper part of the plot (NDM cluster), while TBARS and AGEs levels correlated positively with the DM cluster located at the lower part of the plot (Figure 8 and Table 1).

## 3. Discussion

Diabetes mellitus is a serious, chronic metabolic disease affecting more and more people worldwide. The most recent report of International Diabetes Federation (IDF) shows that there were 463 million of people with diabetes in 2019, and this number may increase to 578 million by 2030 and even up to 700 million by 2045. Hyperglycemia in diabetes results from the inability of the pancreas to produce insulin or lack of effective use of insulin by the body cells [44]. Long-lasting hyperglycemia may be a cause of micro- and macrovascular complications of diabetes, including cardiovascular diseases or renal failure [45]. What is more, diabetes may induce ocular complications, such as accelerated cataract formation [46]. Cataracts are one of the most common visual impairments in diabetics. Nowadays, cataract removal surgery is the main method of treatment. The procedure itself is considered safe, but diabetic patients are more prone to surgery-related complications, such as posterior capsular opacification, postoperative cystoid macular edema, and exacerbation of the diabetic retinopathy [46,47]. Moreover, it should be highlighted that lenses serve not only optical purposes, but are also involved in metabolic processes of the eye. Thus, lens removal may be unfavorable for other eye structures [48]. For this reason, other methods for cataract delay or treatment are being sought, and according to scientific literature, some plant-derived dietary components or nutraceuticals may be helpful [49,50]. Natural compounds may protect the lens through various mechanisms. They might reveal antioxidative and antiglycation properties, inhibit the activity of aldose reductase, and prevent apoptosis of the lens epithelial cells [49]. Berberine demonstrates all these aforementioned mechanisms, which have been demonstrated in numerous in vitro and in vivo studies on various cell lines, tissues, and organs [30,31,37,51,52], which suggests that berberine can be a promising compound protecting the lenses from opacity.

In human clinical studies, berberine is usually used in doses of between 0.5–2.0 g/day [13]. In the present study, we used berberine chloride suspended in water at a dose corresponding to 50 mg/kg of pure berberine. Taking into account the 6.17 conversion factor (which results from the faster metabolism rate in rats) [53], and assuming that average adult person weights 65 kg, the dose used in the present study in rats corresponds to the dose of 0.5 g berberine/day in humans.

In our study, we did not observe any differences in transparency of the examined lenses between the diabetic and non-diabetic rats, but we recorded changes in the majority of the biochemical parameters. Lack of lens opacity after six weeks from streptozotocin injection is not surprising, since other reports indicate that full cataracts in rats develop not earlier than 10–12 weeks after the streptozotocin injection [54,55]. Taking into consideration that one day of life of an adult rat equals about 34.8 days of human life [56], six weeks from the streptozotocin injection corresponds to four human years. The most frequently observed type of cataract in patients with diabetes is the age-related or senile variety, occurring earlier and progressing more rapidly than in healthy individuals [57]. However, the cataract may develop in diabetic children, even when the disease has been present for less than four years [58]. Results of the present study indicate that biochemical changes in rat lenses induced by diabetes may precede the changes in opacity. We assume that such changes should be normalized as soon as possible to prevent the development of cataracts.

We observed that in the lenses of the diabetic rats, there was a significant increase in the activity of aldose reductase (AR), an enzyme catalyzing glucose conversion into sorbitol, the first step in the polyol pathway. It is believed that an intensified polyol pathway and enhanced AR activity is one of the key factors contributing to cataract pathomechanism. Accumulation of sorbitol (and other polyol pathway products) in the lenses disrupts the osmotic equilibrium, leading to swelling and degeneration of the lens fibers and resulting in cataract formation [47]. Similar results with regard to AR activity in the lenses of diabetic rats have also been presented by other authors [55,59,60,61,62]. Administration of berberine to the diabetic rats in our experiment resulted in a significant decrease in AR activity in the lenses. This information seems to be very important and suggests that berberine can protect lenses from osmotic stress and opacity development. In the literature, berberine was described as an AR inhibitor, which reduces AR activity in vitro [63,64,65] and in vivo in the kidneys of rats with streptozotocin-induced diabetes [51].

**Figure A1:**
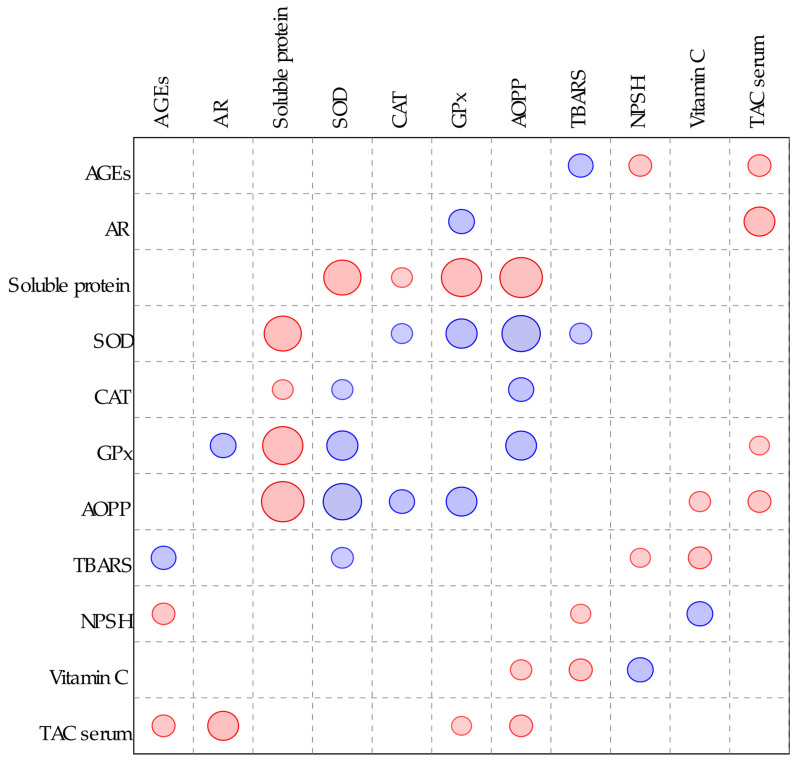
Spearman’s *r_s_* correlation plot of biochemical parameters measured in the lenses and TAC in the serum; blue circles indicate positive correlations, red circles indicate negative correlations, both at *p* < 0.05; circles’ sizes correspond to correlation strength (the bigger the circle, the stronger the correlation). AGEs: advanced glycation end-products; AR: aldose reductase; SOD: superoxide dismutase; CAT: catalase; AOPP: advanced oxidation protein product; TBARS: thiobarbituric acid reactive substance; NPSH: non-protein sulfhydryl group; TAC: total antioxidant capacity.

One of the most important features of diabetes is increased glucose level in the organism. Glucose and its metabolites, such as fructose (the final product of polyol pathway), glyceraldehyde 3-phosphate, and glucose-3-phosphate (intermediate products of glycolysis) interact with the α-amino group of the N-terminal amino acid, or the lysine and arginine ε-amino groups in proteins. This process, called glycation, occurs spontaneously and results in a formation of unstable Schiff’s bases and then Amadori products. Amadori products may undergo further reactions, such as cyclization, oxidation, and dehydration, leading to the formation of stable and irreversible advanced glycation end-products (AGEs)—a heterogenous group of toxic substances [66,67]. AGEs may be also form as a result of lipid peroxidation and glycation [68,69]. Based on the Spearman’s correlation (Figure A1) and principal component analysis (PCA), we observed in our study that AGEs levels correlated positively with TBARS levels—i.e., with the marker depicting the products of lipid peroxidation. In the lenses of the diabetic rats, both the AGEs and TBARS levels were significantly elevated. AGEs can interact with scavenger receptors, such as receptor for advanced glycation end-products (RAGE). When AGEs bind to a RAGE, several signaling pathways are activated, including the Ras/mitogen-activated protein kinases/nuclear factor κB (Ras/MAPK/NF-κB) pathway, which induces NF-κB activation and oxidative stress development [66,67]. In the lenses, AGEs induce irreversible structural changes in the lens proteins (crystallins). Consequently, protein aggregates of high molecular mass are formed, which scatter light and impair the vision. For this reason, the glycation of lens proteins is considered to be another pathomechanism of diabetic cataracts [70]. An observed in the present study increase in the AGEs levels in the lenses of diabetic rats is consistent with literature data [55,71]. We also noted, according to PCA and Spearman’s correlation (Figure A1), that AGEs levels correlated negatively with the level of the non-protein sulfhydryl groups (NPSH). The main compound representing the NPSH in cells is reduced glutathione (GSH) [72,73], which is also the main non-enzymatic antioxidant in the lenses [74]. GSH is described as one of the components responsible for lens transparency, due to the fact that it can bind to the lens proteins protecting their thiol moieties from oxidation [75]. The decrease in NPSH levels in the lenses of diabetic rats observed in our experiment is in accordance with other studies [55,76], and it is an unfavorable change. Plant-derived substances, such as 3,5-di-O-caffeoyl-epi-quinic acid, trans-resveratrol, or hesperetin, are direct inhibitors of AGEs or may enhance the natural mechanisms involved in the detoxication of AGEs and AGE precursors [68]. In the present study, a decrease in the AGEs level in the lenses of the diabetic rats was observed after berberine administration. A lower expression of AGEs and RAGE after berberine treatment has been described previously, with regard to diabetic nephropathy [52]. Berberine is proven to inhibit AGEs formation in vitro [77], but the exact mechanism of how AGEs formation is inhibited by berberine in the lenses of diabetic rats requires further examination. In our study, we did not observe an increase of NPSH after berberine administration. There are also other reports in which NPSH (or specifically GSH) levels remained unchanged in the lenses of diabetic rats after the administration of natural compounds [78,79,80].

In our study, we observed that in the lenses of the diabetic rats, the level of advanced oxidation protein products (AOPP) was significantly elevated compared to the value recorded in the lenses of the non-diabetic animals. Similar to AGEs, AOPP are RAGE ligands, and by interaction with this receptor they contribute to oxidative stress progression [81,82]. AOPP possess in their structures dityrosine cross-links or carbonyl groups, and are markers for protein oxidative damage [83]. In both the principal component analysis and the Spearman’s correlation, AOPP correlated negatively with the level of the soluble protein in the lenses. Therefore, it can be assumed that the elevated AOPP level and reduced level of the soluble protein may be connected with the formation of insoluble protein aggregates in the lenses of the diabetic rats. A decrease in soluble protein levels in the lenses of diabetic animals has been previously reported [55,84]. To the best of our knowledge, there is no available data on the berberine effect on the AOPP level in the lenses or other organs in any experimental model; however, in our previous works we have described that the AOPP level had been reduced in the lenses of diabetic rats treated with different natural substances, such as diosmin, naringenin, chrysin, or resveratrol [79,80,85,86]. Along with the decrease in the AOPP level after administration of berberine to the diabetic rats, an increase in the soluble protein level in this organ was noted. The elevation of soluble protein after treatment with berberine has been also reported in an in vitro study conducted on isolated goat lenses incubated in glucose [39].

In addition to the osmotic imbalance resulting from enhanced polyol pathway and glycation stress, which is manifested by AGEs overproduction, oxidative stress is another contributor to cataract formation in diabetic conditions [87]. In brief, oxidative stress is defined as an imbalance between reactive oxygen species (ROS) production and elimination, with the predominance of the first process [88]. Living organisms possess an antioxidative system that is responsible for neutralizing ROS. This system is complex and the response to oxidative stress is multilevel, and depends on the intensity of this stress. When oxidative stress is low, antioxidative enzymes are synthetized via Keap1/Nrf2-dependent pathway. In oxidative stress at the intermediate level, the activity of antioxidative enzymes is also elevated, but their activation is probably a result of different mechanisms—mainly the NF-κB and activator protein-1 (AP-1) pathways. If the intensity of oxidative stress is low or intermediate, mitogen-activated protein (MAP) kinase and other kinases seem to be involved in signal detection and the induction of cell response, which causes an enhancement of the antioxidative potential. However, if the oxidative stress level becomes high, the overproduced ROS can overwhelm the detoxicating potential of the antioxidative system and lead to apoptotic cell death [88]. The aforementioned mechanisms, and the fact that the reaction to oxidative stress depends on the cellular context that accompanies this stress and the duration of exposition to ROS, can altogether contribute to different responses of antioxidative enzymes in the lenses of the diabetic animals. Some reports indicate that the activity of enzymes like SOD, CAT, or GPx in the lenses of diabetic animals decreases [60,62], while others demonstrate increases in their activity [55,84,85]. This elevated activity of antioxidative enzymes is probably a result of adaptative mechanisms. In a response to the ROS increase, neutralizing antioxidative enzymes are synthesized. SOD converts the superoxide anion (one of the ROS type) into H_2_O_2_ and O_2_, while CAT and GPx remove H_2_O_2_. In our study, the activity of SOD was significantly higher in the lenses of the diabetic rats than in the lenses of the non-diabetic animals. It is suggested that SOD plays an important role in cataract prevention in diabetic conditions; in an experiment conducted on SOD1-null mice, it was shown that the SOD1-lacking animals developed cataracts faster after streptozotocin injection than wild-type mice [89]. It is possible that our study was performed at a time point in which the oxidative stress was at the intermediate level, when the antioxidative systems were still efficient—hence, the lenses were transparent.

Berberine shows low bioavailability after oral administration, due to its low gut absorption and fast metabolism. It is estimated to be 0.68% [3,90]. Low bioavailability of a compound is not necessarily a limiting factor with regard to its beneficial effect on the lenses in rats with experimental diabetes. It has been proven that natural, pharmacologically active compounds have a positive effect on the eye lens despite low bioavailability. For instance, curcumin, whose bioavailability after oral administration is about 1% [91], shows a wide range of beneficial effects on the eye structures, including the eye lens of the diabetic rats [92,93]. Natural substances may also indirectly affect the lens redox system by improving oxidative stress-related parameters in the whole organism. These compounds do not need to be present in the lenses to affect them. Such observations have been made after subcutaneous injection of hesperetin. This flavonoid improved the non-enzymatic markers connected with oxidative stress in the lenses, and reduced the cataract grade in rats; however, it was detected neither in the lenses nor in the serum after 4 h from the injection. The authors of this study concluded that hesperetin acts as an indirect protective agent [94,95]. What is more, it should be noted that several berberine metabolites, such as columbamine, berberrubine, and demethyleneberberine, demonstrate similar pharmacological activities as berberine, and they may be responsible for berberine-related therapeutic effects in vivo [3]. It has been shown that cataract formation is associated with reduced total antioxidative status measured in the serum [96]. We observed that the serum TAC was significantly decreased in the rats with experimentally induced diabetes, while administration of berberine significantly elevated this parameter. This is presumably a result of the antioxidative properties of berberine itself and its metabolites, which are strong antioxidants [28]. Thus, berberine may act as a systemic antioxidant and indirectly protect the lenses from the negative effects of diabetes, especially from AGEs and AOPP formation, as well as AR overactivity (Spearman’s correlation; Figure A1).

In order to comprehensively evaluate the effect of berberine on the biochemical changes in the lenses of the diabetic rats, we performed a multivariate principal component analysis (PCA), in which all tested parameters were used together as a set of variables. This analysis showed that the administration of berberine revealed a beneficial effect on the lenses. Even though some individual parameters, which were analyzed separately in ANOVA, did not differ between the berberine-treated and non-treated diabetic rats, the administration of berberine eventually resulted in overall improvement. There were no statistically significant differences between the clusters formed by the groups of non-diabetic rats and berberine-treated diabetic rats along both PC 1 and PC 2. Moreover, both of these clusters were separated significantly from the cluster formed by the diabetic control rats along PC 1, which explains over 36% of the total variability.

Taking all this into consideration, it can be concluded that oral administration of berberine to diabetic rats for four weeks improves several parameters related to the polyol pathway and glycation processes. This compound reveals antioxidative activity in the serum, but this effect is less visible in the lenses. Nevertheless, this study has several limitations. Firstly, the study was conducted at only one time point (six weeks after the streptozotocin injection), which models the pre-cataract period. Secondly, this experiment was focused only on biochemical changes in the lenses, and morphological and histological analyses were not performed. Therefore, in the future, more detailed studies on the anti-cataract effects of berberine should be conducted, involving morphological and histological examinations as well as genetic assays. These should be performed at more time points, including the time when the formation of cataracts starts and the time when cataracts are fully developed. However, the results obtained in this preliminary study point out the direction of further experiments, elucidating the mechanisms of potential anticataract properties of berberine.

## 4. Materials and Methods

### 4.1. Animals and Experimental Design

The lenses that were examined in the present study were collected during an experiment conducted in the Department of Pharmacology (Faculty of Pharmaceutical Sciences in Sosnowiec, Medical University of Silesia, Katowice, Poland) [97]. All procedures were approved by the Local Ethics Commission in Katowice, Poland (approvals 81/2013–12.11.2013, 114/2014–19.11.2014, 115/2014–19.11.2014).

The study was performed on mature female Wistar rats provided by the Center of Experimental Medicine, Medical University of Silesia (Katowice, Poland). During the two-week acclimation period, as well as during the whole experiment, the rats had an unlimited water supply and were fed with a standard laboratory chow (Labofeed B, Wytwórnia Pasz „Morawski”, Kcynia, Poland). The rats were divided into three groups (*n* = 10 in each group) as follows:

- NDM: non-diabetic, control rats;
- DM: diabetic control rats;
- DM + BRB: diabetic rats treated orally (per os—p.o.) with berberine, at a dose of 50 mg/kg.

The average initial body weight of all rats was 198.4 ± 2.4 g. In the rats from the DM and DM + BRB groups, type 1 diabetes was induced by a single intraperitoneal injection of streptozotocin (Cayman Chemical, Ann Arbor, MI, USA) at a dose of 60 mg/kg. Streptozotocin was freshly dissolved in the citrate buffer (0.1 M, pH 4.5) before use. One week after streptozotocin injection, non-fasting glucose levels in the blood samples collected form tail capillary veins was measured with an Accu-Chek Performa Nano glucometer (Roche Diagnostics GmbH, Mannheim, Germany). Animals with developed diabetes (the blood glucose level above 400 mg/100 mL) were classified to the further steps of the study. Non-fasting glucose was also measured once a week until the end of the experiment. Rats from the NDM group were injected with citrate buffer only.

Two weeks after the streptozotocin injection, the berberine administration started and lasted for four weeks. Berberine, suspended in tap water, was administered by oral gavage. The suspension was prepared in the proportion of 55.3 mg of berberine chloride (i.e., 50 mg of berberine; Sigma-Aldrich, St. Louis, MO, USA) in 2 mL of water, and administered to the rats at a volume of 2 mL/kg. Rats from the NDM and DM groups were treated with tap water at volume of 2 mL/kg.

After four weeks of berberine (or water in the NDM and DM groups) administration, the rats were fasted overnight, and the next day they were euthanized by cardiac exsanguination under general anesthesia (ketamine and xylazine mixture). Before the euthanasia, the fasting blood glucose level was measured. The whole blood collected from the heart was set aside for up to two hours, then centrifuged. The serum total antioxidant capacity (TAC) was evaluated with a commercially available Cayman kit. TAC was expressed as mmol of Trolox eq./L of the serum.

From the euthanized rats, the eyeballs were collected, from which the lenses were separated. The lenses were weighted and homogenized in 10 mM phosphate-buffered saline (PBS), pH 7.4 (10% *w*/*v*), using a glass homogenizer. The obtained homogenate was divided into parts and frozen. The samples were thawed directly before biochemical assays. The total homogenate was used to evaluate thiobarbituric acid reactive substances (TBARS) levels. For other analyses, supernatant obtained from centrifuging at 10,000× *g* for 15 min at +4°C was used. All assays were measured in a Tecan Infinite M200 PRO microplate reader with Magellan 7.2 software (Tecan Austria, Grödig, Austria).

### 4.2. Soluble Protein Level in the Lenses

The supernatant from the lens homogenate, obtained as described above, was used to evaluate the soluble protein level in the lenses. For this purpose, the BioSystems (BioSystems S.A., Barcelona, Spain) kit based on the biuret reaction was used. The level of protein was expressed as mg of protein per 1 g of the lens.

### 4.3. Advanced Oxidation End-Products in the Lenses

The level of the advanced oxidation end products (AGEs) in the lenses was evaluated with the OxiSelect ELISA kit (Cell Biolabs, San Diego, CA, USA). AGEs levels are expressed in µg/g of the lens.

### 4.4. Activity of Enzymes in the Lenses

The activity of aldose reductase (AR) in the lenses was measured by the method described by Hayman and Kinoshita [98], and modified by Halder [99] and Patel et al. [100]. The decrease in absorption was measured at 340 nm for 5 min. The AR activity is presented as nmol of NADPH oxidized/min/mg of protein.

The activities of the antioxidative enzymes, i.e., superoxide dismutase (SOD), catalase (CAT), and glutathione peroxidase (GPx), were evaluated with commercially available kits (Cayman Chemical, Ann Arbor, MI, USA). The activity of SOD is presented as U/mg of protein, where U indicates units of SOD: one U is the amount of enzyme needed to exhibit 50% dismutation of the superoxide radical. The CAT activity is expressed as nmol of formed formaldehyde/min/mg of protein, while GPx is expressed as nmol of NADPH oxidized/min/mg of protein.

### 4.5. Vitamin C and Non-Protein Sulfhydryl Groups in the Lenses

Non-protein sulfhydryl groups (NPSH) levels were assayed according to the Sedlak and Lindsay method, in which 5,5′-dithiobis(2-nitrobenzoic acid) (Sigma-Aldrich, St. Louis, MO, USA) was used [101]. GSH was used for standard curve preparation. NPSH levels are presented as µmol/g of the lens. Vitamin C levels were measured as described in Jagota and Dani [102], using the Folin reagent. The results are presented as µg/g of the lens.

### 4.6. Oxidative Damage Markers in the Lenses

The markers for oxidative damage to the proteins and lipids were measured. For protein oxidation, the advanced oxidation protein products (AOPP) level was assayed as described by Witko-Sarsat et al. [103], using chloramine T as a reference. The results are expressed as nmol eq. of chloramine T/mg protein. A thiobarbituric acid reactive substances (TBARS) assay was used to evaluate the oxidative damage to lipids. This assay was conducted as described by Ohkawa et al. [104], and a standard curve was prepared using 1,1,3,3-tetramethoxypropane. The TBARS levels are presented as nmol/g of the lens.

### 4.7. Statistical Analyses

The results, presented as arithmetical mean ± standard error of the mean (SEM), were subjected to ANOVA followed by Tukey’s honestly significant difference (HSD) post-hoc test in Statistica 12 software (StatSoft Polska, Kraków, Poland). The results were considered statistically significant if *p* < 0.05: * *p* < 0.05, ** *p* < 0.01 and *** *p* < 0.001 refer to statistically significant differences when compared with the NDM group; and ^#^ *p* < 0.05, ^##^ *p* < 0.01 and ^###^ *p* < 0.001 indicate statistically significant differences between the DN + BRB and DM groups. In addition, all the results of the lens biochemical parameters assayed in this study were subjected to principal component analysis (PCA) based on the correlation matrix in the Past 3.21 software [105], and the obtained scores were evaluated by MANOVA in Statistica 12 software. Spearman’s *r_s_* correlations for the biochemical parameters measured in the lenses and the TAC in the serum were calculated in the Past 3.21 software [105] (Figure A1).

## Figures and Tables

**Table 1:** Principal component analysis of biochemical parameters measured in the lenses.

Principal Component	NDM	DM	DM + BRB
PC 1	−1.21 ± 0.38	2.01 ± 0.36 ***	−0.80 ± 0.47 ^###^
PC 2	0.77 ± 0.46	−0.63 ± 0.37 *	−0.14 ± 0.32

The results are presented as an arithmetical mean of PCA scores ± SEM. The statistical significance of the results was evaluated by multivariate analysis of variance (MANOVA) followed by Tukey’s HSD post-hoc test. The results were considered statistically significant if *p* < 0.05: * *p* < 0.05; *** *p* < 0.001—statistically significant differences in comparison with the NDM group; ^###^ *p* < 0.001—statistically significant difference between the DN + BRB and DM groups. NDM: non-diabetic control rats; DM: diabetic control rats; DM + BRB: diabetic rats treated with berberine at a dose of 50 mg/kg for four weeks; PC 1: principal component 1; PC 2: principal component 2.

## Abbreviations

AGEs	Advanced glycation end-products
AOPP	Advanced oxidation protein products
AR	Aldose reductase
CAT	Catalase
DM	Diabetic control rats
DM + BRB	Diabetic rats treated with berberine at a dose of 50 mg/kg p.o.
GPx	Glutathione peroxidase
GSH	Reduced glutathione
NDM	Non-diabetic control rats
NPSH	Non-protein sulfhydryl groups
PCA	Principal component analysis
PC 1, PC 2	Principal component 1, principal component 2
SOD	Superoxide dismutase
TAC	Total antioxidant capacity
TBARS	Thiobarbituric acid reactive substances
